# Purine nucleoside phosphorylase inhibition ameliorates age-associated lower urinary tract dysfunctions

**DOI:** 10.1172/jci.insight.140109

**Published:** 2020-10-15

**Authors:** Lori A. Birder, Amanda Wolf-Johnston, Alan J. Wein, Fangzhou Cheng, Mara Grove-Sullivan, Anthony J. Kanai, Alan M. Watson, Donna Stoltz, Simon C. Watkins, Anne M. Robertson, Diane Newman, Roger R. Dmochowski, Edwin K. Jackson

**Affiliations:** 1 Department of Medicine, Renal-Electrolyte Division, and; 2 Department of Pharmacology and Chemical Biology, University of Pittsburgh School of Medicine, Pittsburgh, Pennsylvania, USA.; 3 Division of Urology, Perelman School of Medicine at the University of Pennsylvania, Philadelphia, Pennsylvania, USA.; 4 Department of Mechanical Engineering and Materials Science, Swanson School of Engineering, University of Pittsburgh, Pittsburgh, Pennsylvania, USA.; 5 Department of Cell Biology, University of Pittsburgh School of Medicine, Pittsburgh, Pennsylvania, USA.; 6 Department of Urology, Vanderbilt Medical Center, Nashville, Tennessee, USA.

**Keywords:** Aging, Cellular senescence

## Abstract

In the aging population, lower urinary tract (LUT) dysfunction is common and often leads to storage and voiding difficulties classified into overlapping symptom syndromes. Despite prevalence and consequences of these syndromes, LUT disorders continue to be undertreated simply because there are few therapeutic options. LUT function and structure were assessed in aged (>25 months) male and female Fischer 344 rats randomized to oral treatment with a purine nucleoside phosphorylase (PNPase inhibitor) 8-aminoguanine (8-AG) or vehicle for 6 weeks. The bladders of aged rats exhibited multiple abnormalities: tactile insensitivity, vascular remodeling, reduced collagen-fiber tortuosity, increased bladder stiffness, abnormal smooth muscle morphology, swelling of mitochondria, and increases in urodamaging purine metabolites. Treatment of aged rats with 8-AG restored all evaluated histological, ultrastructural, and physiological abnormalities toward that of a younger state. 8-AG is an effective treatment that ameliorates key age-related structural and physiologic bladder abnormalities. Because PNPase inhibition blocks metabolism of inosine to hypoxanthine and guanosine to guanine, likely uroprotective effects of 8-AG are mediated by increased bladder levels of uroprotective inosine and guanosine and reductions in urodamaging hypoxanthine and xanthine. These findings demonstrate that 8-AG has translational potential for treating age-associated LUT dysfunctions and resultant syndromes in humans.

## Abstract 2

In a preclinical model, 8-aminoguanine alleviates the functional and structural abnormalities in the aging urinary bladder.

## Graphical abstract

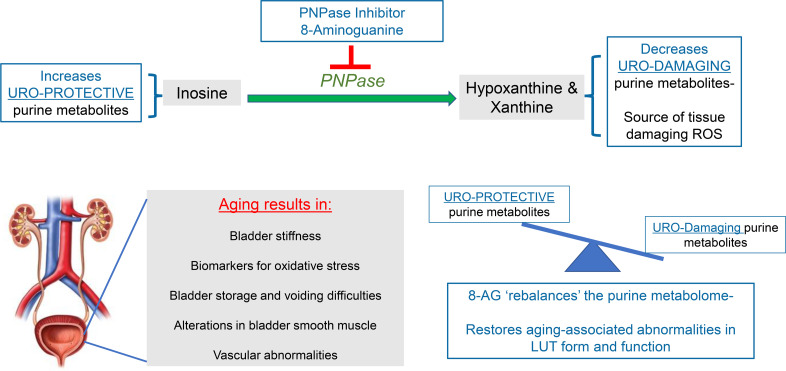

## Introduction

The effects of aging on the lower urinary tract (LUT) are complex (1–3). Multiple bladder components become dysfunctional with age, including the mucosal, muscular, stromal, and neural elements. Pathological features arise from: (a) vascular alterations leading to ischemia and associated reperfusion injury; (b) mucosal alterations characterized by increases in mucosal permeability and loss of mucosal cells, which disrupts cell-cell and cell-interstitium communications; (c) decreased bladder sensation; and (d) the inability of the urinary bladder musculature to exhibit normal compliance (low pressure) during filling and storage and normal contractility during emptying. These pathological differences interact and converge to produce LUT symptoms, including bladder overactivity and urgency, nocturia, impaired bladder contractility, urinary incontinence during filling/storage, a diminished force of urinary stream, and increased residual urine during emptying (4–7). These symptoms are often grouped into overlapping syndromes, such as overactive bladder (OAB) and underactive bladder (UAB), with each set designated as a particular named LUT dysfunction (LUTD) or symptom syndrome. LUTDs are common in the elderly and frail. Indeed, the demographics of LUTDs suggest a steep increase in their occurrence and underlying causative etiologies beginning in the fifth decade in both sexes, increasing until death, and affecting at least 30% of the post–50-year-old population (5, 6).

Despite the prevalence and impact of LUTDs on quality of life in humans, LUTDs continue to be undertreated simply because there are few successful therapeutic options, especially minimally invasive ones, and no preventative strategies nor known modulating interventions that reverse physiologic differences. Emerging evidence, however, suggests that alterations in purine nucleoside phosphorylase (PNPase) levels reflects the participation of oxidative injury and cellular damage (8, 9). Unlike other treatments — such as allopurinol, which targets only hypoxanthine metabolism — blocking PNPase can increase levels of uroprotective precursors (e.g., inosine and guanosine) (10–12), while simultaneously decreasing levels of urotoxic products (e.g., hypoxanthine and xanthine) (13). Complex diseases require pleiotropic medicines to ameliorate the underlying multiple mechanisms that give rise to the pathophysiology. Inasmuch as reactive oxygen species (ROS) are involved in the pathophysiology of aging-related bladder dysfunction and because ROS are produced by multiple pathways, age-related bladder dysfunction does indeed fall into the category of a complex disease that will require pleiotropic drugs to manage. Here we report on our recently developed strategy to correct and reverse age-related differences in bladder dysfunction.

## Results

**Figure 1:**
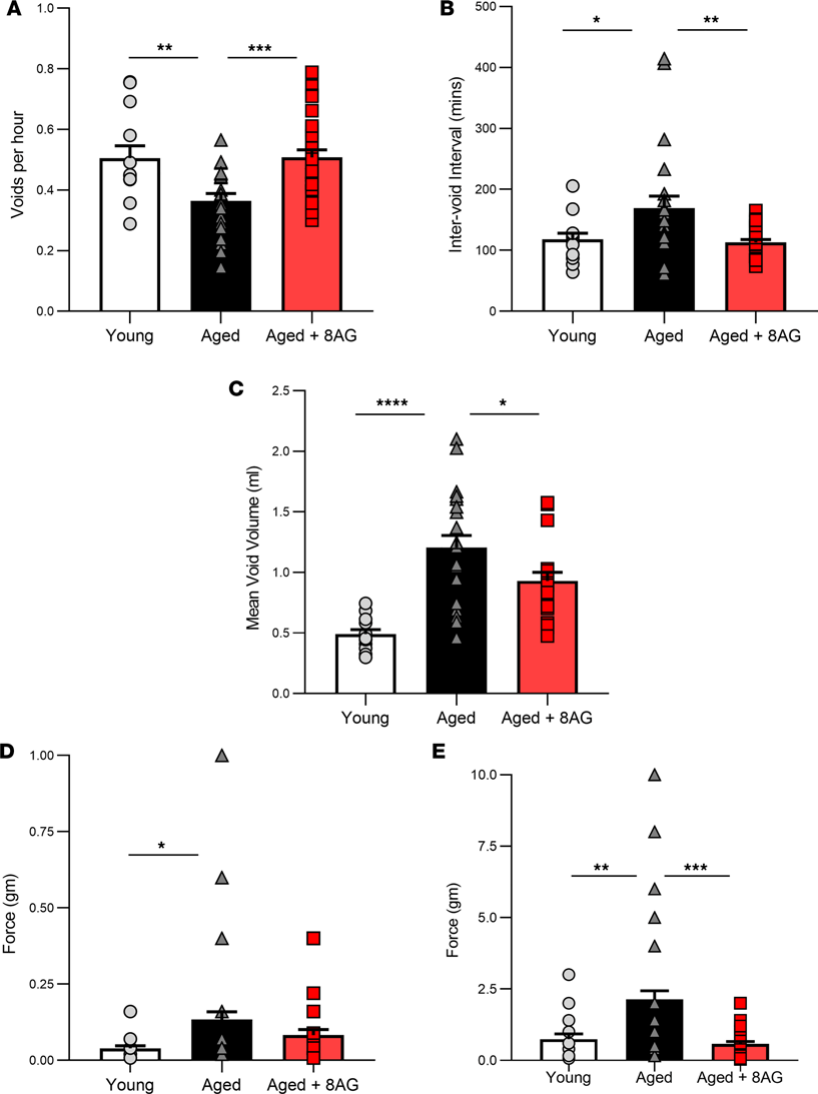
8-Aminoguanine (8-AG) attenuates age-related differences in bladder function. (**A–C**) 8-AG decreases voiding frequency (**A**) (*n* = young, 13; aged, 21; aged + 8-AG, 30) and increases both the inter-void interval (**B**) (*n* = young, 13; aged, 22; aged + 8-AG, 30) and voided volume (**C**) (*n* = young, 13; aged, 21; aged + 8-AG, 22). (**D** and **E**) In addition, aging decreases abdominal (**D**) (*n* = young, 18; aged, 58; aged + 8-AG, 35) and cutaneous (**E**) (*n* = young, 13; aged, 21; aged + 8 AG, 23) responses to tactile mechanical stimuli, which is restored to a younger state by 8-AG treatment. Data are presented as mean ± SEM. Ordinary 1-way ANOVA was used to evaluate significance, which was considered at *P* < 0.05.

Metabolic-cage studies in untreated, aged (>25 months) versus young adult Fischer 344 rats revealed decreases in voiding frequency (Figure 1A) and increases in the intervoid interval (Figure 1B), as well as voided volume (Figure 1C), in aged rats. Also, aged rats demonstrated decreased von Frey sensitivity (14, 15) to tactile (mechanical) stimuli, both abdominal (visceral; Figure 1D) and cutaneous (hind paw; Figure 1E). The von Frey test is a noninvasive behavioral method that uses a series of calibrated von Frey filaments to assess the sensitivity to mechanical stimuli (i.e., response to pressure stimuli) at various anatomical locations. These differences suggest an age-associated deterioration of tactile sensitivity and are suggested to be indicative of sensory decline. Notably, in aged rats treated for 6 weeks with oral 8-aminoguanine (8-AG), both voiding behavior and tactile sensitivity were similar to those observed in young rats.

**Figure 2:**
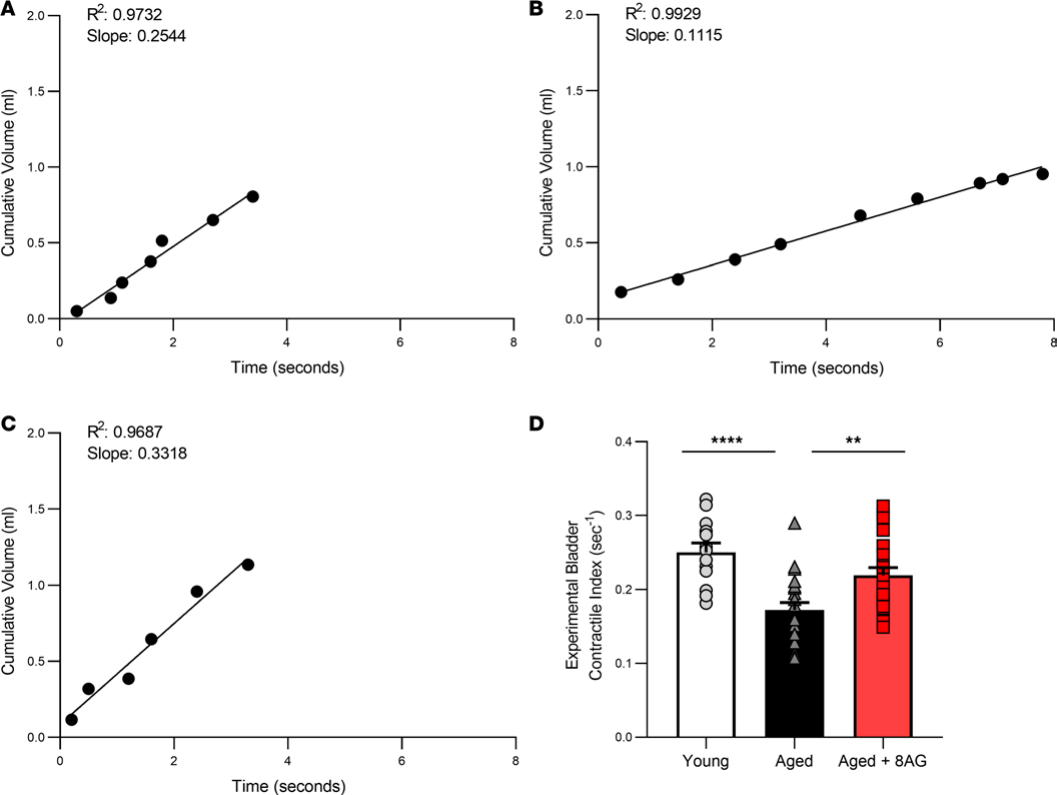
8-Aminoguanine (8-AG) attenuates age-related differences in EBCI. (**A–C**) Representative examples of volume-time relationship plots for young (**A**), aged (**B**), and aged rats treated with 8-AG (**C**). 8-AG also attenuates age-related decreases in EBCI (**D**) (*n* = young, 13; aged, 21; aged + 8-AG, 23).Data are presented as mean ± SEM. Ordinary 1-way ANOVA was used to evaluate significance, which was considered at *P* < 0.05.

The findings illustrated in Figure 1D are consistent with the clinical syndrome designated as UAB. To further investigate whether the aged Fischer 344 rat is an animal model of UAB and whether 8-AG reverses UAB, we sought to develop a more decisive test to diagnose UAB in vivo in rats. It is well known in cardiac physiology that cardiac output is a direct function of preload (i.e., end diastolic volume); thus, a better in vivo estimate of cardiac contractility is afforded by normalizing cardiac output to end diastolic volume (16). We considered that a similar approach would be useful to assess bladder contractility in rats in vivo. Thus, we devised a method to assess bladder activity corrected for bladder “preload,” an approach that yields an Experimental Bladder Contractile Index (EBCI). In this regard, for a given voiding event, the cumulative urine volume (mL) was plotted as a function of time (sec). Each plot was fitted to a straight line using nonlinear regression analysis (without a priori constraints) in GraphPad Prism. The EBCI was calculated by dividing the best-fit slope provided by the regression analysis by the total voided volume. We observed that, for all voiding events, the volume-time relationship was linear (with most *R^2^*-values between 0.95 and 0.99) from the initiation through the completion of voiding (Figure 2, A–C). Therefore, the slope of the best-fit line was used as the most accurate estimate of urine flow rate during a given voiding event. Although urine flow rate is indicative of bladder emptying efficiency, this parameter would be dependent on the initial volume of urine in the bladder (i.e., bladder preload). This is because bladder stretching activates micturition reflexes, which are initiated by local factors released by the bladder cellular elements. To control for preload, for each voiding event, we divided the urine flow rate by total voided urine to give an EBCI. This calculated EBCI was highly reproducible. As shown in Figure 2D, the EBCI in aged rats was significantly lower compared with young rats, a finding consistent with UAB in aged Fischer 344 rats. Importantly, the EBCI in aged rats treated with 8-AG was similar to that of untreated young rats.

**Figure 3:**
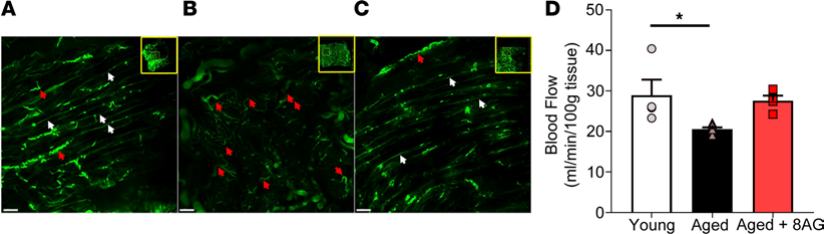
Ribbon-scanning confocal microscopy coupled with perfusion of the bladder with fluorescent beads shows increased vascular tortuosity with appearance of reduced perfusion in aged bladders. (**A** and **B**) Representative image (from *n* = young 3; aged 3 and aged + 8-AG 3 rats) (**B**) showing age-associated differences compared with young bladders (**A**), which demonstrates mostly straight vessels throughout the tissue. (**C**) The aged bladder treated with 8-aminoguanine (8-AG) reduces vessel tortuosity, and the tissue no longer appears ischemic, resembling young tissue. *n* = 9. Panel insets display the reconstruction of the complete bladder with voxels of 0.4 × 0.4 × 10 mm. Yellow boxes display the location of the high-magnification images. Prior to imaging, bladders were cleared using the CUBIC method, and images were acquired by ribbon-scanning confocal microscope. Scale bars: 200 μm (*n* = young 4; aged 6; aged + 8-AG 4). (**D**) Doppler flowmeter measurements revealing a significant decrease in bladder blood flow in aged compared with young rats; blood flow defects in the old bladder are reversed to a younger state with 8-AG treatment. Ordinary 1-way ANOVA was used to evaluate significance which was considered at *P < 0.05.

Bladders perfused with fluorescent beads showed that young bladders contained mostly straight vessels throughout the tissue (white arrows; Figure 3A). In contrast, aged bladders (Figure 3B) displayed only tortuous vessels (red arrows; Figure 3B) and exhibited areas of decreased perfusion that appeared ischemic (severe tortuosity obstructs blood flow and bead injection shows perfusion) (17). Chronic treatment of aged rats with 8-AG (Figure 3C) reduced the number of tortuous vessels, and the tissue no longer appeared ischemic, resembling young tissue. Prior to imaging, bladders were cleared using the CUBIC method (18), and images were acquired by ribbon-scanning confocal microscopy (19). In addition, as shown in Figure 3D, compared with younger rats, untreated aged rats showed a significant decrease in bladder blood flow (measured using a Doppler flowmeter) (20). In contrast, there was no significant difference in bladder blood flow in young versus 8-AG–treated aged rats (Figure 3D).

**Figure 4:**
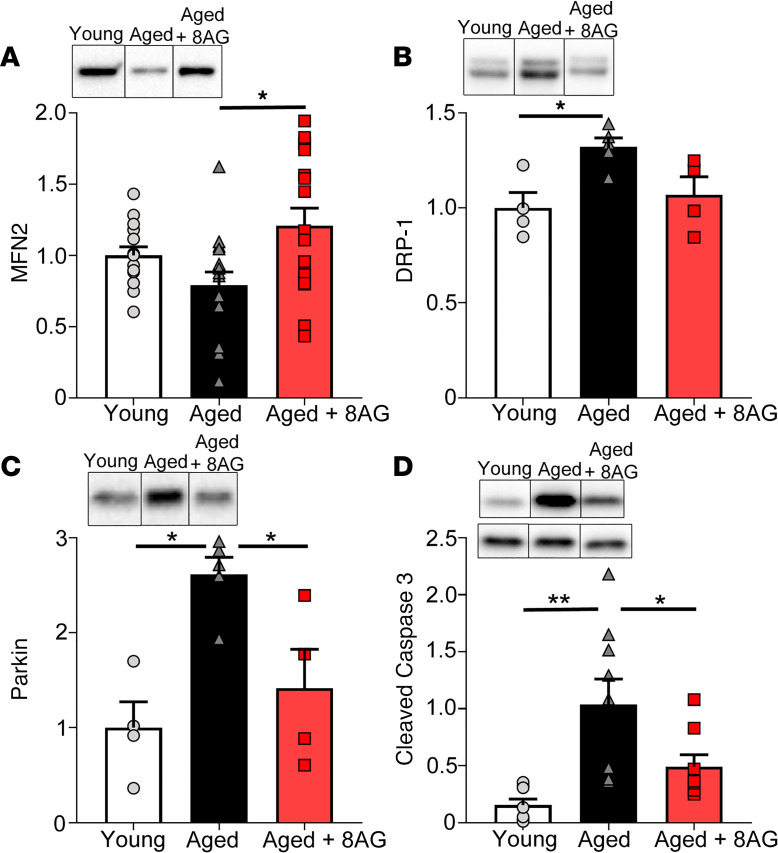
Western immunoblotting revealed significant aging-associated alterations in proteins linked to mitochondrial dynamics and quality control within the bladder mucosa. (**A–D**) Mitofusin 2 (MFN2; *n* = young 14; aged 16; aged + 8-AG 16) (**A**), a protein involved in mitochondrial fusion; Dynamin-|related protein (**B**) (DRP-1; *n* = young 4; aged 5; aged + 8-AG 4), which is involved in mitochondrial fission; Parkin (**C**) (*n* = young 4; aged 5; aged + 8-AG 4), which plays a role in mitophagy; and cleaved caspase-3 (**D**) (*n* = young 8; aged 9; aged + 8-AG 8), which is activated upon initiation of apoptosis**.** In all cases, treatment with 8-aminoguanine (8-AG) restored changes similar to a younger state. Representative immunoblotting is inset within each graph. Cleaved caspase-3 (top of inset) is normalized to total caspase-3 (bottom of inset). All samples were run on the same blot, but representative samples were not contiguous. Data are presented as mean ± SEM. Ordinary 1-way ANOVA was used to evaluate significance. *P < 0.05; **P < 0.01.

As illustrated in Figure 4, A–D, aging was associated with alterations in the expression of key proteins in the bladder mucosa. For example, there was a trend toward decreased mitofusin 2 (MFN2). MFN2 normally facilitates removal of damaged mitochondria and is important for cellular viability by contributing to the maintenance of the mitochondrial network (21). There was also increased dynamin-related protein 1 (DRP-1). DRP-1 is critical for establishing mitochondrial morphology, and overexpression of DRP-1 has been linked to abnormal mitochondrial dynamics and overproduction of defective mitochondria in mammalian cells (22). In the aged rat, Parkin was overexpressed in the bladder mucosa. Parkin has a multitude of functions, including removal of damaged mitochondria under conditions of oxidative stress (23). Finally, in aged rats, caspase-3 expression was elevated in the bladder mucosa. Caspase-3 is a protein that promotes apoptosis (24). With 8-AG treatment, these biochemical abnormalities in the bladder mucosa were restored to levels similar to those of the younger state.

**Figure 5:**
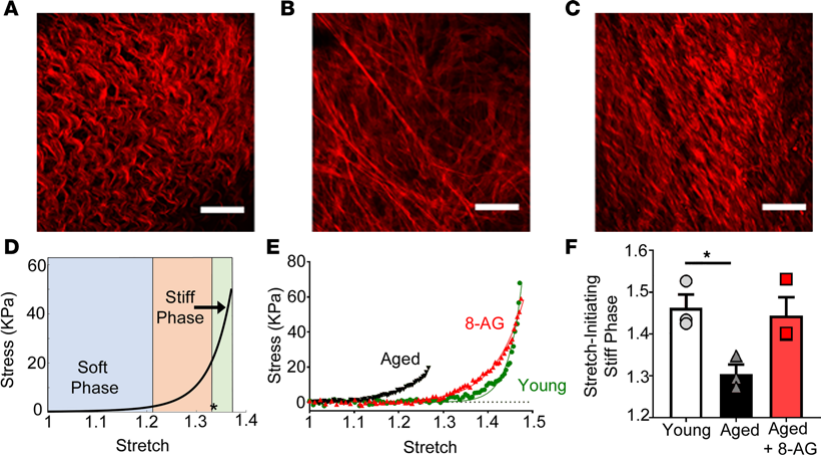
Concurrent imaging and mechanical testing of bladder collagen fibers in the detrusor layer of young versus aged bladder. (**A**) The collagen fibers in young bladder are highly tortuous (occurred in 3 of 3 rats) enabling large stretch with very little change in load (stress). (**D)** This soft phase of the stress-stretch curve is followed by a transition to a stiff phase at higher stretch (asterisk indicates onset of stiff phase or critical stretch). (**B**) There is a substantial decrease in tortuosity in the detrusor layer of aged bladders (occurred in 3 of 3 rats) compared with young bladders, leading to a premature recruitment of collagen fibers. (**E**) The early recruitment leads to shortening of the soft phase and early shift to the stiff phase in aged versus young bladders (best fit graph with individual data points plotted; *n* = 3 each for young, aged, aged + 8-AG). (**C–F**) This trend was reversed after 8-aminoguanine (8-AG) treatment, resulting in partial recovery in tortuosity (**C**) (occurred in 3 of 3 rats), rightward shift of stress-stretch curve toward the young bladder curve (**E**), and recovery in critical stretch for onset of stiff phase (**F**). Data are presented as mean ± SEM. Scale bars: 100 mm. Ordinary 1-way ANOVA was used to evaluate significance. *P < 0.05.

In all bladders, the passive response of bladder wall specimens to mechanical loading under prescribed stretch was measured as a surrogate for the pressure/volume relationship seen during bladder filling (25) (representative graph from *n* = 12 rats, Figure 5E). In all bladders, the mechanical reaction to mild stretch was a compliant response (soft phase) in which large changes in the extension of the bladder wall caused little change in stress. Upon further stretch, a noncompliant response (stiff phase) was reached (Figure 5D), in which small changes in the extension of the bladder wall caused a large change in stress. The stretch at which this steep increase in stiffness occurred (the critical stretch, indicated by an asterisk in Figure 5D) depended on the tortuosity of the collagen fibers in the detrusor layer. In this regard, the tortuosity enabled bladder filling at low mechanical loads (or pressure) because fibers contributed little to load bearing until they were straightened (recruited). As increasing numbers of fibers were recruited, the bladder rapidly stiffened. For the aged bladder, collagen fibers in the detrusor layer were recruited early (Figure 5B) relative to the young bladder at the same stretch (Figure 5A). This early recruitment was caused by the diminished collagen-fiber tortuosity in the aged bladder and resulted in a leftward shift of the bladder’s stress-stretch relationship (Figure 5E), manifested as a substantial reduction in the stretch at which the stiff phase for bladder occurred in aged relative to young rats (Figures 5E). Notably, after 8-AG treatment, the aged bladder regained the collagen-fiber tortuosity in the detrusor layer (Figure 5C), and this manifested as recovery of the prolonged soft or compliant regime (i.e., rightward shift of the stress-stretch relationship) seen in young bladders (Figure 5E). Correspondingly, the critical stretch for the treated aged bladder recovered, approaching that of the young bladder (Figure 5F), also seen as a rightward shift of the stress-stretch relationship (Figure 5E).

**Figure 6:**
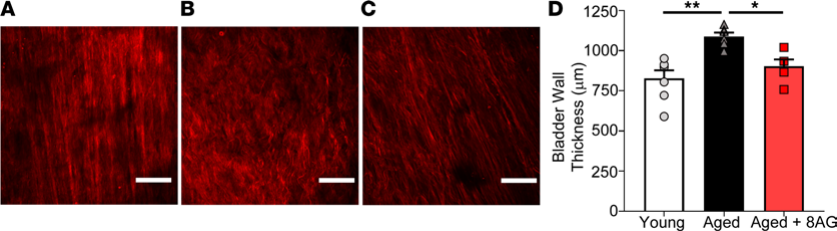
Concurrent multiphoton imaging and biomechanical testing of bladder collagen fibers in lamina propria of young versus aged rat bladder. (**A**) The collagen fibers in the lamina propria of young bladder are recruited (straightened) under physiological loading levels (occurred in 3 of 3 rats). (**B**) In contrast, the aged bladder did not expand sufficiently to recruit collagen fibers, even at physiological loads (occurred in 3 of 3 rats). (**C**) Collagen fibers in the bladders of aged animals treated with 8-AG were able to be recruited (straightened) at physiological loading levels (occurred in 3 of 3 rats). Aged bladders also exhibit increased wall thickness as compared with younger rat bladders. (**D**) Bladder wall thickness in old rats was restored toward a younger state with 8-AG treatment (*n* = young, 4; aged, 4; aged + 8-AG, 4). Data are presented as mean ± SEM. Scale bars: 100 mm. Ordinary 1-way ANOVA was used to evaluate significance. *P < 0.05; **P < 0.01.

As a result of the premature stiffening in the detrusor, the untreated aged bladder never expanded sufficiently to effectively recruit collagen fibers in the lamina propria layer, even at physiological levels of bladder stretch (Figure 6B). In contrast, the collagen fibers within the lamina propria layers of both the young and treated aged bladders were able to contribute to load bearing at physiological loading levels (Figure 6, A and C). Taken together, these data support the conclusion that treatment of older rats with 8-AG restored collagen fiber tortuosity in the detrusor layer (Figure 5C), corresponding to a larger fraction of highly tortuous fibers, which would allow increased filling at a lower pressure. This resulted in a more extensible (i.e., compliant) bladder closer to a normal state. In addition, bladders from aged rats also exhibited a significant increase in bladder wall thickness (Figure 6D), which was restored to that of a younger state by 8-AG treatment.

**Figure 7:**
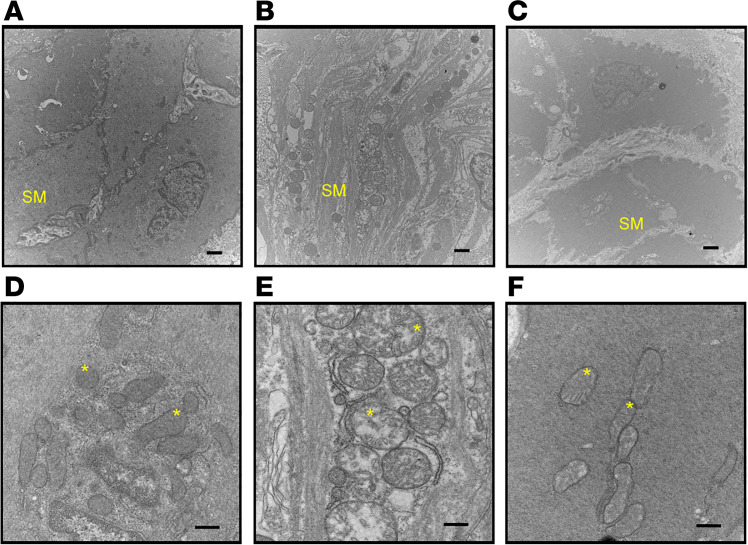
Representative transmission electron microscopy images of bladder smooth muscle in young rats, aged rats, and aged rats (*n* = 3 each) treated with 8-aminoguanine (8-AG). (**A** and **B**) These images reveal abnormal detrusor smooth muscle (SM) morphology in aged rats. Panel **B** includes separation and degeneration of cells as compared with young rats (**A**) (3 of 3 rats). However, the abnormal morphology in aged rats is restored to a younger state by 8-AG treatment (**C**; 3 of 3 rats). (**D–F**) Higher-magnification transmission electron microscopy images revealing substantial swelling and disruption of smooth muscle mitochondria (asterisk denote mitochondria) in aged bladders (**E**; 3 of 3 rats) compared with young bladders (**D**; 3 of 3 rats); these anomalies were restored to a younger state by 8-AG treatment (**F**; 3 of 3 rats). Scale bars: 1 µm (magnification, 15,000×) (**A–C**) and 600 nm (magnification, 30,000×) (**D–F**).

Aging was also associated with significant morphological differences in the urinary bladder smooth muscle showing a separation and degeneration of smooth muscle cells (compare Figure 7A with Figure 7B). Furthermore, this structural pathology was accompanied by swelling and disruption of the smooth muscle mitochondria (compare Figure 7D with Figure 7E). With 8-AG treatment, smooth muscle structural anomalies (Figure 7C), including mitochondrial alterations (Figure 7F), were reversed.

**Figure 8:**
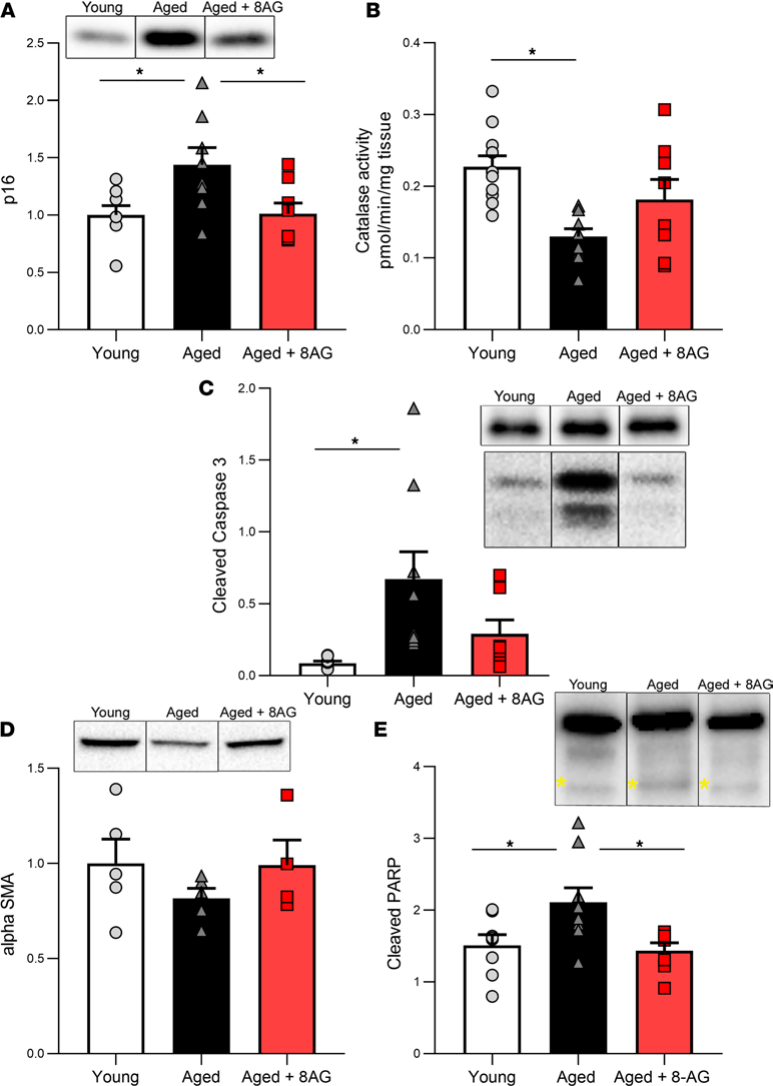
Aged bladder detrusor exhibited alterations in various biomarkers. (**A–C**) Senescent biomarker p16 (**A**) (*n* = young, 8; aged, 8; aged + 8-AG, 8), catalase activity (**B**) (*n* = young, 11; aged, 10; aged + 8-AG, 8), and cleaved caspase-3 (**C**) (cleavage product shown in lower panel [17-19kDa], compared with uncleaved caspase-3 in upper panel; 35kDa; *n* = young, 8; aged, 9; aged + 8-AG, 7). (**D**) Aged bladder detrusor smooth muscle exhibit alterations in α-smooth muscle actin (*n* = young, 5; aged, 5; aged + 8-AG, 4). (**E**) Significant differences were deterred in PARP activation, as indicated by cleavage product at 89 kDa (lower band, yellow asterisks), compared with uncleaved PARP (upper band, 116 kDa) (*n* = young, 8; aged, 9; aged + 8-AG, 7). 8-AG treated in aged rats restored these biomarkers to those of a younger state. Representative immunoblotting is inset within each graph. All samples were run on the same blot, but representative samples were not contiguous. Data are presented as mean ± SEM. Ordinary 1-way ANOVA was used to evaluate significance. *P < 0.05.

In addition, aging was associated with a trend toward decreased expression of the smooth muscle marker α-smooth muscle actin (Figure 8D) and significant increases within detrusor smooth muscle in both the cellular senescence marker p16 (Figure 8A) and in catalase activity (Figure 8B), which plays a role in defenses against ROS in aging (26). We also observed a significant increase with aging in both cleaved caspase-3 (Figure 8C) and cleaved PARP (Figure 8E) (27), both of which are involved in senescence and programmed cell death. Moreover, 8-AG restored the expression of the senescence marker p16 (Figure 8A), catalase activity (Figure 8B), cleaved caspase-3 (Figure 8C), and cleaved PARP (Figure 8E) to levels similar to the younger state.

**Figure 9:**
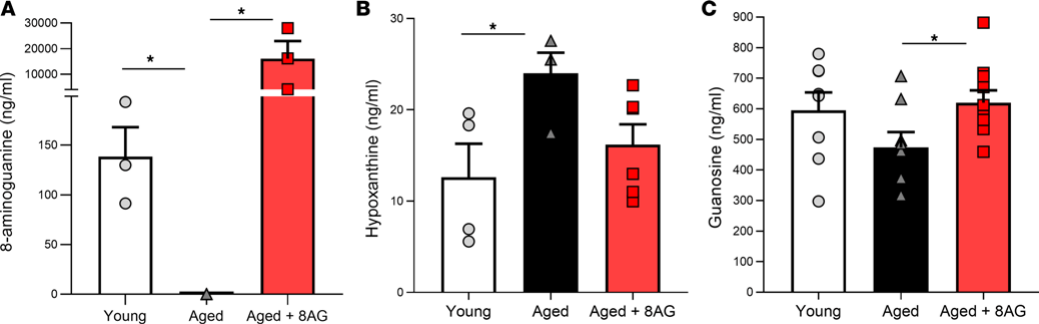
Purine metabolome measurements in young, aged, and aged rats treated with 8-aminoguanine. (**A**) In aged rats, endogenous urinary 8-aminoguanine (8-AG) is below assay detection limits (*n* = young, 3; aged, 4; aged + 8-AG, 3). (**B**) However, aged rats have higher urinary hypoxanthine levels (*n* = young, 4; aged, 4; aged + 8-AG, 6); both of these abnormalities are restored to younger levels with 8-AG treatment. (**C**) In addition, guanosine levels (*n* = young, 8; aged, 8; aged + 8-AG, 10) are altered with age and recovered with 8-AG treatment. Ordinary 1-way ANOVA was used to evaluate significance. *P < 0.05.

PNPase transforms inosine and guanosine to their respective bases (i.e., inosine into hypoxanthine and guanosine into guanine) (8, 9, 28). In untreated aged rats, urinary levels of endogenous 8-AG were undetectable but were restored to younger levels following oral 8-AG treatment (Figure 9A). Furthermore, in untreated aged rats, urinary hypoxanthine levels were higher compared with young rats, and oral 8-AG treatment reduced urinary hypoxanthine to levels comparable with younger rats (Figure 9B). Aged rats exhibited a trend toward decreased urinary levels of guanosine (protective role in age-related diseases) (29), yet urinary guanosine levels were similar in 8-AG–treated aged rats compared with young rats (Figure 9C).

## Discussion

Here, we report results comparing urinary bladder form and function in young, aged, 8-AG–treated aged rats. Our findings demonstrate that untreated older rats, compared with young rats, exhibit (a) bladder filling/storage and emptying dysfunction, (b) decreases in tactile sensitivity to mechanical stimuli, (c) abnormal bladder vascular remodeling and reduced bladder blood flow, (d) abnormalities in smooth muscle morphology and mitochondrial structure, (e) reduced collagen fiber tortuosity, (f) decreased bladder compliance, (g) increased urinary levels of urodamaging hypoxanthine, and (h) decreased urinary levels of 8-AG. Importantly, all LUT outcome measures were similar in young rats versus older rats treated with 8-AG, an endogenous and potent inhibitor of PNPase. These findings demonstrate that the PNPase inhibitor 8-AG has strong translational potential for treatment of age-associated LUTDs in humans.

PNPase, not to be confused with PNPT1, is an enzyme that is expressed in most tissues (28). This enzyme belongs to the family of glycosyltransferases, is expressed in both bacteria and mammals, and is one of the key enzymes involved in the purine salvage pathway (30, 31). PNPase transforms inosine and guanosine to their respective bases (i.e., inosine into hypoxanthine and guanosine into guanine) (8, 9, 28). Patients with a complete lack of PNPase activity have a decreased T cell function, resulting in a disorder of the immune system termed immunodeficiency (32). Thus, this enzyme is involved in cell metabolism of nucleosides and nucleotides, and helps maintain immune function.

Although PNPase is a critically important enzyme in the purine salvage pathway, there are reasons to hypothesize that partially inhibiting PNPase might protect the LUT from injury. In this regard, both inosine and guanosine exert beneficial antiinflammatory and tissue-protective effects in various target organ systems, including the LUT. For example, administration of exogenous inosine exerts protective effects on the urinary bladder following experimentally induced obstruction or spinal cord injury (10, 11). The mechanism of action may involve stimulation of adenosine receptors and/or prevention of oxidative damage via scavenging of free radicals and peroxynitrite. Guanosine is also tissue protective in a number of animal models of injury or degenerative diseases. In contrast to guanosine and inosine, elevated levels of inosine’s downstream metabolite hypoxanthine over time may exhibit harmful effects due to production of ROS when metabolized by xanthine oxidase to xanthine. The increased metabolism of hypoxanthine is linked to inflammatory and other disorders, including ischemia (13). For example, a shift in purine catabolism with enhanced accumulation of (potentially injurious) hypoxanthine may play a role in declining myocardial tolerance to ischemia with aging (33). Increased oxidative damage by ROS is deleterious to cells and plays a key role in progression of a number of diseases. Because hypoxanthine is a potential free-radical generator, hypoxanthine has also been used as an indicator of hypoxic conditions. Not surprisingly, treatments that inhibit oxidation of hypoxanthine suppress inflammatory cytokines and oxidative stress in a number of disorders. Because PNPase inhibition blocks the metabolism of inosine to hypoxanthine and guanosine to guanine, the uroprotective effects of PNPase inhibitors in general — and 8-AG in particular — are likely mediated by increases in bladder levels of inosine and guanosine (uroprotective purines) and reductions in bladder levels of hypoxanthine (urodamaging purine and ROS generator). However, it should be noted that, in addition to hypoxanthine-associated ROS expression, 8-AG may exert beneficial effect on bladder function by multiple pathways, including those that impact immune function and inflammation. Unlike other treatments such as allopurinol (which only targets hypoxanthine metabolism), blocking PNPase should increase uroprotective precursors (inosine and guanosine) while simultaneously decreasing levels of urotoxic hypoxanthine.

Despite the ability of PNPase inhibitors to increase inosine and guanosine while decreasing hypoxanthine, we did not anticipate that 8-AG would have such wide-ranging beneficial effects on molecular, cellular, and functional abnormalities in the bladder. Inosine, guanosine, and hypoxanthine have not been previously shown to affect microvascular architecture, reorganize collagen fibers, or reverse mitochondrial abnormalities. Also, the wide-ranging effects of 8-AG in aging are surprising. Our studies were performed in rats near the end of their life span and who had already developed severe bladder pathologies that were unlikely to be reversed by any treatment. However, 8-AG treatment for only 6 weeks completely or partially reversed all of the measured molecular, cellular, and functional bladder abnormalities associated with aging. The efficacy of 8-AG to reverse age-related decrements in bladder form and function may be due to pleiotropic effects associated with blocking PNPase.

Although 8-AG was quite effective with regard to reversing age-related bladder pathologies, one must be cognizant of potential toxicities associated with this strategy. PNPase deficiency, inherited as an autosomal recessive disorder, leads to build up of deoxyguanosine triphosphate, which is toxic to dividing cells. However, even though PNPase is ubiquitously expressed, the deficiency of this enzyme specifically affects T cells, which predisposes patients to repeated and persistent infection (34). In addition, infants with PNPase deficiency have been shown to grow more slowly as compared with healthy infants (35). Thus, given this background, one would expect that inhibiting PNPase would be too toxic to treat bladder disorders. However, studies show that partial inhibition of PNPase is safe. For example, heterozygous parents with only 1 normal PNPase gene have one-fourth to half the normal activity of PNPase yet are not affected by the deficiency. Moreover, nearly complete inhibition of PNPase with forodesine is necessary to suppress the purine salvage pathway (36, 37).

The LUT is particularly susceptible to the negative effects of age (and intercurrent morbidities), with increased prevalence of storage and voiding symptoms with aging (1–3, 5, 6). Thus, it was imperative that we evaluate bladder function in vivo. In this regard, a recent study compared a number of functional LUT measurements in rats assessed by metabolism-cage experiments versus urodynamic measurements; the results were indistinguishable (38). The main advantage of this approach was that key variables could be assessed in conscious rats. Another advantage was that measurements could be performed without causing cellular damage (caused by catheterization) so that subsequent ultrastructural, biochemical, and molecular studies could be reliably performed.

Our findings that both bladder filling/storage and emptying are significantly impaired with age is consistent with clinical reports of UAB in older patients (2, 39–41). In addition, our findings of an increase in intervoid interval, as well as voided volume, are in line with findings using aged rodents (42). This suggests that aging may be, in part, associated with a change in the structure or function of smooth muscle and/or a decrease in sensitivity to differences in bladder volume. We compared bladder structure and function in young adult versus aged rats. Since studies were not conducted on animals with a range of ages, in the present study, differences with respect to bladder function and cellular state between young adult and aged rats could have been due to either aging per se or, alternatively, to maturation processes. Indeed, it would be highly informative to evaluate bladder function and structure throughout the life span of Fischer 344 rats.

In the present study, we captured in vivo voiding events in conscious rats with second-by-second time resolution. This acquisition of fine-granular data allowed us to explore in detail the relationship between voided volume and time in conscious rats. We were impressed by the remarkably linear relationship between cumulative voided volume and time, and we exploited this to develop a potentially novel approach for assessing voiding efficiency — i.e., the EBCI. The EBCI provides an index that reflects bladder voiding efficiency (as measured by the slope of the volume-time relationship assessed by regression analysis) normalized to bladder preload (as estimated by the total void volume). Since the vigor of bladder emptying is driven by volume-sensitive (stretch-sensitive) neural reflexes and release of autocrine/paracrine factors, the EBCI provides an estimate of bladder efficiency that can be compared among rats with different initial bladder preloads. This methodology provided supporting evidence that, (a) in conscious, aged rats, voiding efficiency is indeed reduced — a finding consistent with clinical observations in older patients and that (b) 8-AG corrects age-related reductions in voiding efficiency.

While data on aging and bladder filling sensation is limited, studies suggest that alterations in both bladder afferent structure and function with advanced age can impair urinary bladder storage and voiding. Age-associated differences in sensation may be due to anatomical and functional alterations in peripheral nerves, in addition to decreased blood flow to nerve endings and other risk factors (such as nerve damage from chronic diseases such as diabetes) (43). In general, the elderly are less sensitive to mechanical stimuli and exhibit reduced ability to detect vibration, touch, and pressure (44–46). Our findings of age-associated deficits in both abdominal (visceral) and tactile (cutaneous) sensory functions support this view. While the underlying mechanism for these differences may involve a number of factors, studies in animals have shown that increased oxidative stress (47) may be a contributing factor to aging of the peripheral nervous system (48). Our findings of a reversal of tactile and abdominal sensory function with 8-AG (which decreases damaging purine metabolites such as hypoxanthine a source of ROS) supports a role for oxidative damage.

Older patients exhibit a number of alterations with age, such as decreased smooth muscle cell function and increased fibrosis or stiffening of the bladder (decreased compliance) that can lead to an increase in urinary urgency and/or inability to empty the bladder well. For example, an ultrastructure study focusing on the bladder detrusor by Elbadawi et al. showed that patients with geriatric voiding dysfunction (e.g., impaired contractility) exhibited distinctive smooth muscle cell atrophy and degeneration (49). Consistent with findings in older adults, we show that aged rat detrusor smooth muscle exhibit structural alterations, including smooth muscle degeneration, swelling, and disruption of the mitochondria and abnormalities of a number of key proteins associated with mitochondrial quality control. At the cellular level, mitochondria are considered the powerhouse of organelles, generating 95% of all cellular energy; they are major players in energy production and intracellular communication and are associated with a number of age-related diseases (47, 50–52). Mitochondrial decline is known to be one of the key hallmarks of aging and age-related disorders. In this regard, mitochondrial dysfunction has been shown to be associated with increased oxidative damage and differences in mitochondrial morphology and function (53, 54). In further support of the critical role that mitochondria play in aging are studies that show that increasing mitochondrial function or decreasing mitochondrial ROS production extends the health span in multiple species.

Aging-related alterations in the extracellular matrix (ECM) may also impact the function of various cell types in the bladder wall. Despite having different etiologies, most chronic fibrotic disorders are associated with a persistent production of similar factors, including ROS, that stimulate excessive tissue remodeling (e.g., ECM production), which progressively destroys the organ’s architecture and, in turn, its function (55, 56). As the bladder fills, the coordinated recruitment of collagen fibers across both the smooth muscle and lamina propria layers, essential for the elasticity of the bladder wall, is lost during aging. Furthermore, this impacts the ability of the urothelium to sense changes in mechanical deformation occurring during a micturition cycle and release mediators that may influence sensation. Both collagen and elastin networks compose the majority of the ECM of most organs, including the skin, and also provide structural support; there is limited evidence that increased collagen fiber accumulation may occur in bladders of older patients (49, 57). However, our study focused on mechanisms underlying how aging can impact collagen architecture, which in turn influences functional properties of the bladder. Not only is the amount of these fibers important for proper bladder function, but their orientation, conformation, and recruitment during bladder filling are also of importance. For example, collagen type III fibers (found in the walls of distensible organs and blood vessels) display specific orientations depending on bladder volume, as well as the specific location of the fibers within the bladder wall (58). When the bladder is quiescent and empty, fibers appear as loose (wavy) networks with random orientation. During bladder expansion, fibers straighten in the direction of the applied force and appear long and thin, lying parallel to the urothelium and the smooth muscle. This arrangement likely allows maximal bladder storage without imposing stress on the bladder wall, thus assuring adequate bladder compliance and minimizing bladder injury.

Our findings reveal that aging leads to decreased recruitment of collagen fibers during bladder stretch, which is likely to correlate with increased bladder stiffness; these differences are restored to that of a younger state by 8-AG, suggesting a link between oxidative stress and changes in ECM stiffness. In aging bladders, changes in the smooth muscle/collagen ratio and possibly ECM composition likely impair the orientation, conformation, and even recruitment of collagen fibers. Our group has previously shown, using a combination of biaxial stretch and multiphoton imaging, that a coordinated recruitment of collagen across the lamina propria and detrusor layers is essential for normal elasticity of the bladder wall (25). As in our prior work, we found that wall compliance can be lost in the aging bladder by premature recruitment of collagen fibers. These fibers effectively arrest further expansion of the bladder. This suggests that an altered tension/stretch may be imposed on the urothelial cells and/or smooth muscle as the bladder fills. This modified mechanical stress on the urothelium and smooth muscle likely alters mechanical sensing and impairs paracrine communication within the bladder wall; thus, it may contribute to bladder symptoms reported in aged adults.

Elevated levels of hypoxanthine may exhibit harmful effects to the LUT due to the production of ROS when hypoxanthine is metabolized by xanthine oxidase to xanthine and then to uric acid. It is known that hypoxanthine, via transporters ENT1 and -2 (59, 60), is efficiently transported across cell membranes; therefore, increased urinary hypoxanthine can gain access to underlying tissues, leading to cellular and organ damage. Our findings reveal that urinary hypoxanthine levels are elevated in aged rat bladder, as well as in urine from a limited number of patients with LUTDs as compared with controls without underlying urologic disorders (unpublished observations). Thus, though ROS can have physiologic roles, sustained ROS levels (for example, in aging and age-related disorders) are likely to result in tissue injury due to oxidative damage and mitochondrial dysfunction — ultimately resulting in diminished sensation, cellular loss/damage, and excessive deposition of collagen fibers/loss of elasticity.

Symptoms common in this group of older patients include poor bladder contraction during emptying with decreased stream and residual urine (UAB), OAB during filling/storage (often coexisting with UAB during emptying), and varying types of urinary incontinence or loss of bladder control (stress, urgency, or mixed or spontaneous incontinence), arising from a combination of bladder and urethral dysfunction. The demographics of these disorders suggest a steep increase in occurrence of these symptoms and their underlying causative etiologies beginning in the fifth decade in both sexes, increasing until death, and affecting at least 30% in aggregate of the post–50-year-old population. While patients and health care providers may consider these conditions a normal part of aging, clearly LUTDs are not normal and impair quality of life and burden health care resources (61).

Treatments for LUTDs include behavioral therapy and pelvic floor exercises, antimuscarinics, α-adrenoceptor agonists, electrical stimulation, and interventional therapies (e.g., periurethral bulking agents, suburethral support tapes, and artificial urinary sphincters for stress urinary incontinence, and onabotulinum toxin-A injections into the bladder wall for overactivity) (62–65). However, these treatments, which are largely unsuccessful, are frequently not adequate to control or reverse the symptoms, and many have a high incidence of adverse events. For example, drugs such as the antimuscarinics have been associated with the development of cognitive deficiencies in the elderly, intradetrusor onabotulinum toxin-A can result in urinary retention, and artificial urethral sphincter implantation carries a high incidence of infection or malfunction (66). Thus, despite the prevalence and consequences of LUTDs, many of these conditions continue to be undertreated. Clearly, treatment of LUTD is an unmet medical need with limited to no available options. No current nor historic therapies are known to prevent the advent of the above noted histologic or physiologic abnormalities, so the promise of an effective treatment that would avert the consequences of age and injury-related bladder dysfunction is extremely enticing.

### Conclusions.

It is clear from the above discussion that (a) age-related LUTDs are prevalent and, given the aging of a substantial segment of every society, are increasingly so and (b) there is an unmet need for effective and safe treatments for LUTDs. While efficacy in comparison with other purine scavengers is not known, our long-term rat studies (unpublished observations) with 8-AG demonstrated lack of toxicity to the heart, liver, kidney, brain, and adrenal gland as assessed by histological examination of these tissues following 40 days of 8-AG treatment. In this context, our previous finding that 8-AG treatment is safe and our current findings that 8-AG ameliorates the molecular, cellular, and functional abnormalities in the aging bladder are particularly important, perhaps offering hope to millions who suffer from life-altering LUTDs. These findings may indicate broad-field differences throughout the urinary tract, which could portend a parallel extensive symptoms–based responsiveness in a variety of urinary syndromes that are highly prevalent and have substantial impact on quality of life.

## Methods

### Animals.

This study employed male and female young, yet mature (3 months old) and aged (25–30 months old) Fischer 344 rats (Charles River Laboratories and the National Institute on Aging [NIA] rodent colony). Aged rats were treated with oral 8-AG (5 mg/kg/day for 6 weeks in drinking water; Toronto Research Chemicals) versus a control (untreated) group. We noted no differences between 8-AG or control rats in either food or water intake, body weight, or overall behavior. We also observed no difference between males versus females tested, and since we did not observe a robust difference in signal between the two, we combined the data.

### Voiding analysis.

Young, aged, and 8-AG–treated aged rats were placed in metabolic cages (24 hours; once per week) for the duration of the study, and an average of all measurements was obtained for each rat. The light cycle was from 7 a.m. to 7 p.m., and food and water were provided ad libitum. Voided urine was collected in cups attached to force displacement transducers (Grass Technologies) connected to a computer (WinDaq data acquisition software DATAQ Instruments Inc.). Data were averaged for 24 hours and also analyzed for 12-hour periods during the day (7 a.m.–7 p.m.) and night (7 p.m.–7 a.m.). Voiding frequency (voids per hour), intervoid interval, and volume per void were analyzed. Voiding frequency was calculated as the number of voiding events per hour during 24 hours and during the 12-hour day and 12-hour night periods. Volume per void, which defines bladder capacity, was calculated as an average of the voids occurring during these periods. Voiding efficiency was estimated for a given voiding event by plotting the relationship between cumulative void volume (mL) and time (seconds). Each plot was fitted to a straight line using nonlinear regression analysis (without a priori constraints) in GraphPad Prism. The EBCI was calculated by dividing the best-fit slope provided by the regression analysis by the total voided volume. Although the total voided volume does not take into account residual urine in the bladder, it does provide a readily captured estimate of the bladder preload.

### von Frey testing.

Forty-eight hours after the metabolic cage studies, tactile sensitivity was measured once per week for the duration of the study (an average of all measurements was obtained for each rat) using von Frey nylon filaments (Stoelting Co.) applied to the suprapublic and plantar hind paw (14, 15). von Frey thresholds have been used for pain sensitization as well as age-associated differences in sensitivity to mechanical or pressure stimuli. The withdrawal threshold (using up-down method) was determined, with positive withdrawal responses defined as sharp retractions, licking/scratching, or vocalizations, and the threshold was defined as the force (grams) represented by the von Frey filament, which elicited the positive response.

### Vascular alterations.

Bladders that had been perfused transcardially with PBS buffer containing 20 nm yellow-green fluorescent beads (Thermo Fisher Scientific, F8787) to highlight the vasculature were fixed and then cleared by removing lipids and dissolving light-absorbing chromophores within the tissue (18). Volumetric data were acquired using a Caliber ID RS-G4 ribbon scanning confocal microscope (Caliber ID), which can acquire large-area images at high (350 nm) resolution using a high NA 1.0, 20× long working distance (8mm) lens (CFl190 20xc GLyc; NIKON Inc.; uses a high nA [1.0] long working distance [8 mm] with low magnification [20×]) (19). This affords the advantage of interrogating whole tissues in a 3-dimensional environment without the need to section. Real-time blood perfusion (1 mm^3^ tissue) was accomplished using a BLF22D laser Doppler flowmeter (Transonic Systems Inc.) with a surface probe (TypeS-APLPHS) applied to the serosal surface of the bladder (apex and neck) wall using Doppler light shift from moving RBCs to analyze flow by the Bonner algorithm. This method gives robust, noninvasive microvascular flow signals in the bladder wall of anesthetized rats.

### Biaxial stretch combined with multiphoton microscopy (MPM) imaging.

Planar biaxial mechanical testing coupled with multiphoton microscopy was performed on bladder wall specimens to assess bladder mechanical function while simultaneously observing changes in the collagen microstructure (25). Methods followed those in ref. 25. Briefly, within 2 hours of harvest, intact bladders (*n* = 9 including 3 aged, 3 aged treated, and 3 young) were cut open longitudinally and trimmed into 6 ± 1 mm × 6 ± 1 mm square specimens for mechanical testing with sides aligned in the in situ longitudinal and circumferential directions. Samples were then positioned in our custom-designed biaxial testing system and mechanically tested. Local stretch was calculated using fiducial markers on the sample, defined as the ratio of the distance between markers in the sample when loaded and unloaded. Stress was calculated based on the load measurement and initial cross-section area perpendicular to the loading direction. During testing, samples were immersed in HBSS without calcium and with added EDTA (0.5 mM), nifedipine (5 μM; MilliporeSigma), and thapsigargin (1 μM; Tocris Biosciences) to inhibit smooth muscle cell contraction.

A multiphoton microscope (Olympus FV1000 MPE) equipped with a Coherent Cheleon TiSapphire pulsed Laser was used to image the undulated (tortuous or wavy) collagen fibers in the mounted samples without staining or fixation during loading (i.e., stretch) as per our previously published methods (25). Stacks of 2D planar images were generated by imaging sequentially across the wall thickness. To avoid tissue damage while obtaining a large range of stretch, loading was stopped at the stretch where collagen fibers were visibly straightened (heretofore termed recruited). This was defined as the maximum stretch. Fiber tortuosity was measured by tracing collagen fibers across the 2D slices (Filament function in Imaris) (25, 67). Fiber arc length and cord length were determined for each fiber tracing and used to calculate the tortuosity. The tortuosity of an undulated or wavy fiber is therefore greater than 1 and approaches 1 as the fiber becomes fully straightened.

### Transmission electron microscopy (TEM).

Urinary bladder sections from each group (young, aged, and aged + 8-AG treatment) was fixed in cold 2.5% glutaraldehyde in 0.01M PBS. The specimens were rinsed in PBS, post-fixed in 1% osmium tetroxide with 1% potassium ferricyanide, rinsed in PBS, dehydrated through a graded series of ethanol and propylene oxide solutions, and embedded in Poly/Bed 812 (Luft formulations). Semithin (300 nm) sections were cut on a Leica Reichart Ultracut, stained with 0.5% Toluidine Blue in 1% sodium borate, and examined under the light microscope. Ultrathin sections (65 nm) were stained with uranyl acetate and Reynold’s lead citrate and examined on a JEOL 1400 transmission electron microscope with a side mount AMT 2k digital camera (Advanced Microscopy Techniques).

### Western immunoblotting.

Bladder preparations were homogenized using Lysing Matrix D in a FastPrep 24 instrument (MP Biomedicals) in HBSS (5 mM KCl, 0.3 mM KH_2_PO_4_, 138 mM NaCl, 4 mM NaHO_3_, 0.3 mM Na_2_HCO_3_, 0.3 mM Na_2_HPO_4_, 5.6 mM glucose, and 10 mM HEPES, pH 7.4) containing complete protease inhibitor cocktail (1 tablet/10 ml, Roche) and phosphatase inhibitor cocktail (MilliporeSigma, 1:100). After centrifugation (16200*g*, 15 minutes at 4°C), the membrane protein fraction was prepared by suspending the membrane pellets in lysis buffer containing 0.3M NaCl, 50 mM Tris-HCl (pH 7.6), and 0.5% Triton X-100, as well as the same concentration of protease inhibitors as above. The suspensions were incubated on ice and centrifuged (16200*g*, 15 minutes at 4°C). The protein concentrations of the combined supernatants were determined using the Pierce BCA protein assay (Thermo Fisher Scientific). After denaturation (100°C for 5 minutes) in the presence of Laemmli sample buffer, lysate from each sample was separated on a 4%–15% TGX Stain-Free SDS-PAGE gel (Bio-Rad). As a reliable loading control, total protein measurement per sample was determined using Bio-Rad Stain Free SDS-PAGE gel technology. UV-activated protein fluorescence was imaged on a ChemiDoc MP (Bio-Rad). After proteins were transferred to polyvinylidene fluoride membranes, the membranes were incubated in 5% (w/v) dried milk dissolved in TBS-T (20 mM Trizma, 137 mM NaCl, 0.1% Tween-20, pH 7.6), rinsed with TBS-T, and incubated overnight at 4°C with primary antibodies — including α-smooth muscle actin (Novex, 701457), senescent marker p16 (Abcam, AB51243), MFN2 (Abcam, AB56889), DRP-1 (Cell Signaling Technologies, 8570), Parkin (Cell Signaling Technologies, 4211), caspase-3 (Cell Signaling Technologies; cleaved 9664, total 9665), and PARP (Cell Signaling Technologies, 9542), diluted in TBS-T containing 5% (w/v) milk. After washing in TBS-T, the membranes were incubated with secondary antibodies — sheep anti–mouse HRP (Southern Biotech) or donkey anti–rabbit HRP (Advansta) for 1 hour in 5% (w/v) Milk TBS-T, washed, and incubated in WesternBright Quantum (Advansta). They were then imaged on a ChemiDoc MP (Bio-Rad). The volume (intensity) of each protein species was determined and normalized to total protein using Image Lab software (Bio-Rad).

### Purine metabolome measurement.

Urine samples were diluted 1 to 30 with water, and heavy isotope internal standards were added to each sample. Purines were separated by reversed-phase ultraperformance liquid chromatography (Waters UPLC BEH C18 column, 1.7 μm beads; 2.1 × 150 mm) and quantified by selected reaction monitoring using a triple quadrupole mass spectrometer (TSQ Quantum-Ultra; Thermo Fisher Scientific) with a heated electrospray ionization source. The mobile phase was a linear gradient flow rate (300 μL/min) of 1% acetic acid in water (pH 3; mobile phase A) and 100% methanol (mobile phase B) and was delivered with a Waters Acquity ultraperformance liquid chromatographic system. The gradient (A/B) settings were: from 0 to 2 minutes, 99.6%/0.4%; from 2 to 3 minutes, 98.0%/2.0%; from 3 to 4 minutes, 85.0%/15.0%; and from 4 to 6.5 minutes, 99.6%/0.4%. The instrument parameters were: sample tray temperature, 10°C; column temperature, 50°C; ion spray voltage, 4.0 kV; ion transfer tube temperature, 350°C; source vaporization temperature, 320°C; Q2 CID gas, argon at 1.5 mTorr; sheath gas, nitrogen at 60 psi; auxiliary gas, nitrogen at 35 psi; mass resolution of quadrupoles 1 and 3 was 0.7 units full-width half-maximum; scan width, 0.6 units; scan time, 0.01 seconds. The following transitions (selected reaction monitoring) were obtained: guanosine (284 → 152 *m/z*, retention time [RT] = 3.10 min); ^13^C10^15^N_5_-guanosine (299 → 162 *m/z*, RT = 3.10 min); hypoxanthine (137 → 119 *m/z*, RT = 1.86 min); ^13^C_5_-hypoxanthine (142 → 124 *m/z*, RT = 1.86 min); aminoguanine (167 → 150 *m/z*, RT = 1.50 min); and ^13^C_2_^15^N-aminoguanine (170 →153 *m/z*, RT = 1.50 min).

### Statistics.

Data were analyzed in GraphPad Prism 6 using Student’s *t* test (1 tailed) and 1-way ANOVA followed by appropriate post hoc tests. *P* < 0.05 was considered significant. Results are expressed as means ± SEM. *P < 0.05; **P < 0.01.

### Study approval.

The IACUC of the University of Pittsburgh approved all procedures. The investigation conforms to the *Guide for the Care and Use of Laboratory Animals* (National Academies Press, 2011).

## Author contributions

LAB, SCW, AMR, and EKJ designed research studies. AWJ, FC, MGS, AMW, and DS conducted experiments. AWJ, FC, MGS, AMW, and DS acquired data. LAB, SCW, AJK, AMW, DS, AMR, DN, AJW, and EKJ provided reagents and developed methodologies. LAB, SCW, AMR, AJW, RRD, and EKJ wrote the manuscript. LAB, SCW, AMR, AJW, RRD, and EKJ interpreted data.

## Version 1 09/10/2020

In-Press Preview

## Version 2 10/15/2020

Electronic publication
